# Estimating effective pressures in active subglacial lakes with ICESat-2 satellite altimetry

**DOI:** 10.1017/jog.2025.10116

**Published:** 2025-12-17

**Authors:** Aaron Stubblefield, Aleah Nicholson Sommers, Colin Meyer, Lauren Cristy Andrews

**Affiliations:** 1 Earth System Science Interdisciplinary Center, University of Maryland, College Park, MD, USA; 2 Global Modeling and Assimilation Office, NASA Goddard Space Flight Center, Greenbelt, MD, USA; 3 Thayer School of Engineering, Dartmouth College, Hanover, NH, USA

**Keywords:** glacier hydrology, laser altimetry, subglacial lakes, subglacial processes

## Abstract

The difference between the ice and water pressures, or the effective pressure, influences water flow and sliding at the ice-bed interface. Effective pressure is typically quantified with subglacial hydrology models because direct measurements of the subglacial environment are sparse. Active subglacial lakes provide an opportunity to constrain effective pressures with altimetry because subglacial water-volume changes manifest at the ice-sheet surface as elevation-change anomalies. Here, we develop a method for estimating effective pressures from altimetry data above active subglacial lakes. We synthesise a previous theory of subglacial lake effective pressure with an altimetry-based inverse method that relates elevation-change data to water-volume changes. We apply the method to elevation-change data from NASA’s ICESat-2 satellite altimetry mission over several active lakes in Antarctica. We find that deviations from flotation (zero effective pressure) are typically a negligible fraction of the overburden (e.g., 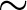
10 kPa), although larger deviations can arise when the ice viscosity is large. For example, effective pressures over subglacial lake Byrd_s10_ in East Antarctica locally reached magnitudes on the order of the tensile strength of glacier ice (e.g., over 100 kPa). These effective pressure estimates can constrain subglacial hydrology models in regions with active subglacial lakes and provide new insights into glacier-bed dynamics.

## 1. Introduction

Subglacial water flow is regulated by the effective pressure, the difference between the ice and water pressures at the glacier base (Röthlisberger, 1972; Shreve, 1972). The effective pressure controls rates of creep closure in subglacial drainage elements such as channels and cavities, and thereby influences the volumetric discharge by restricting water flow (Nye, 1976; Flowers, 2015). Likewise, the magnitude and direction of water flow are determined by hydraulic potential gradients, which depend on gradients in the effective pressure (Hewitt, 2011). The effective pressure also influences the frictional behaviour at the ice–bed interface and modulates ice–flow speeds (Lliboutry, 1968; Bindschadler, 1983; Schoof, 2005; Zoet and Iverson, 2020). While the effective pressure is a fundamental variable that controls subglacial water flow and sliding at the ice–bed interface, direct measurements obtained via borehole drilling are sparse (Iken and others, 1993; Fountain, 1994; Hubbard and others, 1995; Engelhardt and Kamb, 1997; Meierbachtol and others, 2013; Andrews and others, 2014; Rada and Schoof, 2018).

Subglacial lakes that are observed to episodically fill and drain, often called ‘active’ lakes, present an opportunity for constraining effective pressures with altimetry data because subglacial water-volume changes manifest at the ice-sheet surface as elevation-change anomalies (Fricker and others, 2007; 2010; Fricker and Scambos, 2009; Smith and others, 2009). In particular, the coupling between the effective pressure in a subglacial lake with the surrounding drainage system drives the water-volume oscillations that are expressed at the ice-sheet surface (Evatt and others, 2006; Fowler, 2009; Stubblefield and others, 2019). The ICESat (2003–09) and CryoSat-2 (2010 to present) satellite altimetry missions have detected over one hundred active lakes beneath the Antarctic Ice Sheet, while a smaller number have been found in Greenland, Iceland and various mountain glaciers (Smith and others, 2009; Wright and Siegert, 2012; Siegfried and Fricker, 2018; Livingstone and others, 2022). NASA’s ICESat-2 satellite altimetry mission (2018 to present) has allowed for continued detection and monitoring of active subglacial lakes at high spatial and temporal resolution (Neckel and others, 2021; Siegfried and Fricker, 2021; Livingstone and others, 2022; Fan and others, 2023; Freer and others, 2024; Gray and others, 2024a; 2024b).

Previous modelling work quantified subglacial lake effective pressures with a finite element method (Stubblefield and others, 2021b). A limitation of the finite element method is that the mean effective pressure in the lake is determined numerically with a Lagrange multiplier, which only furnishes an indirect relation between lake activity and viscous ice flow. An alternative approach was developed to estimate subglacial water-volume changes with an inverse method that accounts for the effects of viscous ice flow on surface-elevation changes (Stubblefield and others, 2023a). The inverse method assumes that subglacial lake oscillations represent small perturbations to an ice-flow state that is described by auxiliary parameters such as the ice thickness, viscosity, flow speed and basal sliding coefficient (cf. Gudmundsson, 2003).

In this study, we develop a method for estimating effective pressures from elevation-change anomalies above active subglacial lakes by synthesising the previous modelling approaches (Stubblefield and others, 2021b; 2023a). First, we derive general expressions for the effective pressure that depend on the ice–surface elevation and viscous ice flow. Then, we relate the effective pressure to the elevation change and basal vertical velocity with a linearised model (Stubblefield and others, 2023a). We present semi-analytical and synthetic examples to illustrate the basic behaviour of the method. Finally, we apply the method to a collection of subglacial lakes in Antarctica that have shown activity during the ICESat-2 era.

## 2. Model derivation

**Figure 1.**
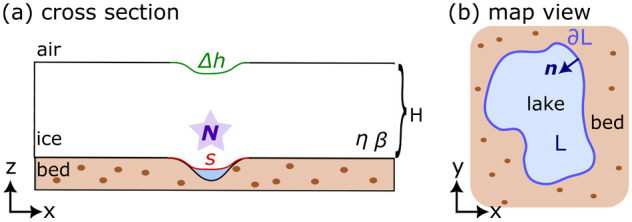
(a) Sketch of a subglacial lake in cross-section highlighting the elevation-change anomaly 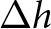, ice-base elevation 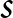, and effective pressure 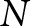. The ice layer over the lake is characterised by the thickness 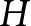 and viscosity 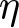, while the ice-bed interface is characterised by the basal drag coefficient 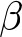. (b) Map-view sketch showing the lake area 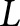, lake boundary 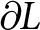 and normal vector 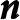.

**Figure 2.**
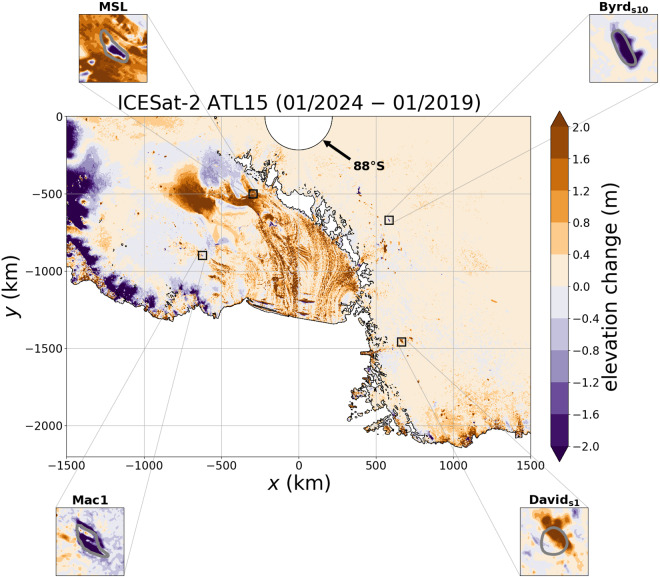
Map of ICESat-2 elevation-change data from Antarctica (ATL15 gridded product; Smith and others, 2024) with insets showing anomalies over subglacial lakes Mac1 (MacAyeal Ice Stream), Mercer Subglacial Lake (MSL; Mercer Ice Stream), Byrd_s10_ (Byrd Glacier) and David_s1_ (David Glacier). The map-plane 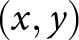 coordinates in the ATL15 dataset correspond to the Antarctic Polar Stereographic Projection (EPSG:3031). Lake outlines from Siegfried and Fricker (2018) are shown in silver on the insets.

In this section, we first derive general formulas for the effective pressure, which is defined by
(1)
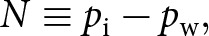


where 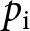 is the ice pressure and 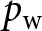 is the water pressure. We refer to the condition 
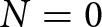
—when the ice and water pressures are balanced—as ‘flotation’. Then, we use a linearised (small perturbation) model to relate the effective pressure in a subglacial lake to the basal vertical velocity anomaly 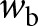 that produces the observed surface-elevation change 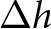 (Figs. 1,2).

We represent the ice-surface elevation 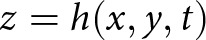 via 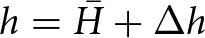 where 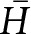 is the background ice thickness and 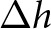 is the elevation-change anomaly over the lake (Fig. 1a). The lower surface of the ice is denoted by 
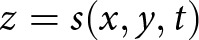
. We assume that ice deforms as a viscous fluid according to the incompressible Stokes equations with a linear sliding law at the base and a stress-free condition at the ice-air interface (Stubblefield and others, 2023a). For consistency with the linearised framework, we assume a uniform ice viscosity 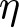, basal drag coefficient 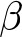 and ice thickness 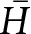 in the vicinity of the lake. The assumptions of a linearised framework and uniform material properties are adopted because subglacial lakes are expected to only generate small changes in ice flow relative to a given background state (Stubblefield and others, 2023a). The primary limitation of this approach is that the basal drag coefficient does not depend on the time-varying effective pressure in the subglacial lake, but is instead assumed to be constant. The relevant components of the linearised model are summarised below while precise details on the derivation are outlined in Appendix A.

### 2.1. Effective pressure formulas

#### 2.1.1. Hydrostatic approximation

We derive an expression for the effective pressure in a subglacial lake by assuming that the water pressure follows a hydrostatic gradient,
(2)
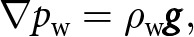


where 
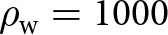
 kg m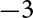 is the density of water and 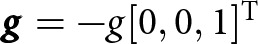 is gravitational acceleration with magnitude 
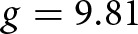
 m s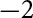. The water pressure is not known *a priori* because it depends on the stresses in the overlying ice column (Stubblefield and others, 2021b). Therefore, the water pressure is determined up to a constant that depends only on the map-plane coordinates 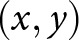, which we resolve by averaging over the surface area of the lake. In this way, we obtain a formula for the water pressure at the ice–water interface (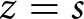),
(3)
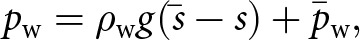


where bars denote spatial means over the surface area of the lake (Stubblefield and others, 2021b). Taking the difference of the ice pressure 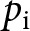 with (3), we obtain an expression for the effective pressure 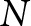 over the subglacial lake,

(4)
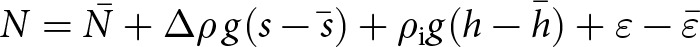

(5)
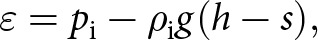

where we have defined the density difference, 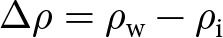, and the difference between the ice pressure and cryostatic pressure, 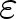. Two challenges arise in applying the formula (4) to estimate effective pressures in subglacial lakes. First, the mean effective pressure (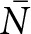) and the deviation of the ice pressure from the cryostatic pressure (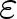) are undetermined at this stage, requiring incorporation of the ice-flow dynamics (Stubblefield and others, 2021b). Second, the motion of the ice–water interface 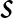 does not necessarily correspond to the motion of the ice surface 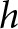 (Stubblefield and others, 2021a; 2023a). We resolve these challenges by directly incorporating the effects of ice flow into the method below.

The hydrostatic assumption implies that subglacial lakes are still bodies of water that tend to capture water from surrounding areas. Gradients in the subglacial lake effective pressure (4) balance the ‘background’ hydraulic gradient of the subglacial drainage system in the limit of a cryostatic ice pressure,
(6)
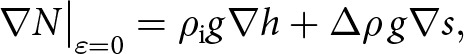


which implies that the water flux is zero and that the lake corresponds to a minima in the hydraulic potential (e.g., Hewitt, 2011). In subglacial drainage systems, water flows due to deviations from the background hydraulic gradient, so we cannot estimate effective pressures with the formula (4) outside of the lake boundary. Therefore, we will restrict formulas to the interior of the lake below by introducing an indicator function 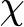, defined by
(7)
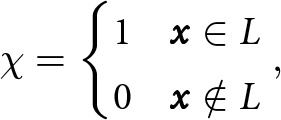


where 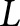 denotes the lake area (Fig. 1). While subglacial lake shorelines can migrate during volume-change events, we assume a fixed lake boundary 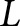 for simplicity (Siegfried and Fricker, 2021; Stubblefield and others, 2021a; 2021b). We revisit this assumption in the discussion. Extending the current implementation to allow for time-varying boundaries (evolving 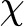) would be straightforward (see Data Availability statement for code repository).

#### 2.1.2. Small-slope approximation

We derive a direct relation between the effective pressure and ice dynamics by considering the normal stress at the ice-water interface. Assuming that the slope of the basal surface 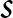 is small over the subglacial lake, the normal stress 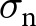 at the base 
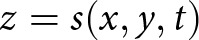
 is given by
(8)
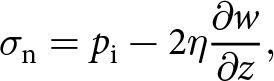


where 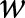 is the vertical component of the ice velocity (Stubblefield and others, 2023b). The normal stress equals the water pressure at the ice-water boundary, which implies that the effective pressure is given by
(9)
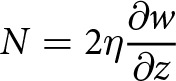


at 
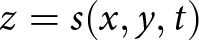
. Incompressibility leads to an expression in terms of the horizontal flow divergence,
(10)
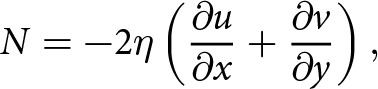


where 
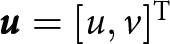
 is the horizontal velocity. Eqn. (10) shows that the effective pressure is controlled by viscous flow towards the lake, reflecting the concept of creep closure. The ice is floating (
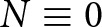
) when the horizontal flow divergence vanishes over the lake as in the case of rigid motion or cryostatic balance. An approximate analysis using (10) and mass continuity shows that the effective pressure is related to dynamic thickness changes through the logarithmic strain rate of the ice column (Appendix B). The effective pressure formulas (9)-(10) are general in the sense that they hold everywhere there is an ice–water interface with small slope. Below, we combine the effective pressure formula (9) that is based on viscous ice stresses with the hydrostatic formula (4) to estimate effective pressures in active subglacial lakes.

We integrate Eqn. (10) over the lake area 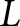 and use the divergence theorem to obtain an expression for the mean effective pressure,
(11)
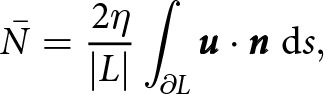


where 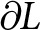 is the boundary of the lake, 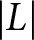 is the surface area of the lake, and 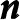 denotes an inward-pointing unit normal to the lake boundary (Fig. 1b). In previous work, the mean effective pressure 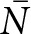 was determined with a Lagrange multiplier, which provided an indirect relation between the effective pressure and ice flow (Stubblefield and others, 2021b). Eqn. (11) instead provides a direct relation between the mean effective pressure in a subglacial lake and viscous flow towards the lake.

As a simple example, Eqn. (11) implies that a lake with circular boundary has a mean effective pressure of 
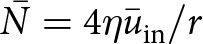
 where 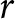 is the radius of the lake and 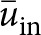 is the average inflow speed. This simple expression shows the sensitivity of the mean effective pressure to the ice viscosity. In the limit of a large lake area (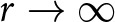), the mean effective pressure approaches zero analogous to a floating ice shelf (Stubblefield and others, 2023b). On the other hand, the mean effective pressure can become large in the limit of a small lake area (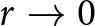), although the inflow speed 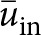 may also diminish in this limit. While lakes that are smaller than the ice thickness are difficult to detect with altimetry-based methods (Stubblefield and others, 2021a), many small lakes that could have elevated effective pressures have been detected with ice-penetrating radar (Wright and Siegert, 2012; MacKie and others, 2020).

### 2.2. Perturbation formulas

Next, we introduce a linearised (small perturbation) approach for estimating the effective pressure 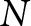 from elevation changes (Stubblefield and others, 2023a). We assume a uniform ice thickness 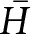 in the base state where the elevation of the ice-water interface 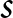 corresponds to 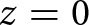 (Fig. 1). The viscosity 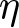 and basal drag 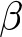 are also assumed to be uniform in the vicinity of the lake. The limitations of these assumptions have been discussed and tested against a fully nonlinear model in previous work (Stubblefield and others, 2023a). Formally, we consider 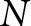, 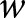, 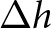 and 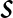 to be perturbations to a cryostatic base state characterised by the ice pressure 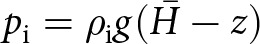 in addition to vanishing vertical velocity and effective pressure fields. In the base state, Eqn. (8) implies that the mean water pressure is cryostatic (
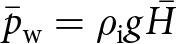
) while the mean effective pressure is zero (
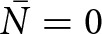
). Likewise, the parameter 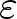 vanishes in the cryostatic base state. We test the assumption that the background state is at flotation (
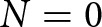
) below by considering dynamic thickness changes away from the lake (Table 1; Appendix B).

We linearise the effective pressure around the base-state lower-surface elevation (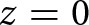) via
(12)
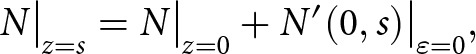


where the second term represents the directional derivative of 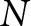 at 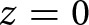 in the direction of the ice–water interface perturbation 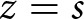 in the cryostatic base state (
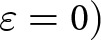
. We find from Eqn. (4) that the directional derivative is 
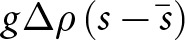
. On the other hand, we resolve the first term in (12) with the expression (9) evaluated at 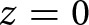.

With these considerations, the linearised expression (12) becomes
(13)
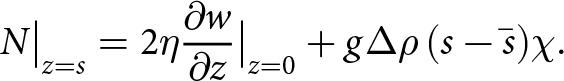


The first term in (13) captures the effects of viscous ice flow while the second term represents perturbations to the hydrostatic normal stress due to movement of the ice base (cf. Stubblefield and others, 2023b). The second term (directional derivative) in (13) has been truncated with the indicator function 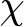 because variations in the effective pressure cannot be constrained outside of the lake boundary with the hydrostatic formula. In particular, we recover the expression for the mean effective pressure (11) from Eqn. (13) because the mean of the hydrostatic term over the lake area vanishes.

**Figure 3.**
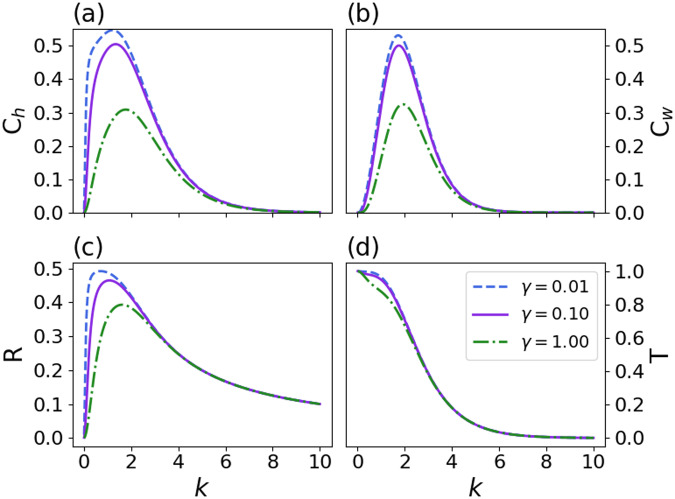
(a,b) Functions 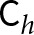 and 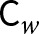 (Eqns. 15-16) that determine the Fourier-transformed effective pressure 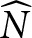 (Eqn. 18) as functions of the scaled wavevector magnitude 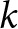 for different values of the nondimensional parameter 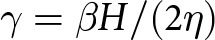. (c,d) Functions 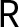 and 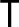 (Eqns. 23-24) that determine the Fourier-transformed elevation-change anomaly 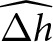 (Eqn. 22) for different values of 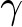. All functions are nondimensional.

We solve the linearised problem by deriving a formula for 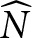, the Fourier transform of the effective pressure (13) with respect to the map-plane coordinates 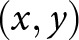. In Appendix A, we show that
(14)
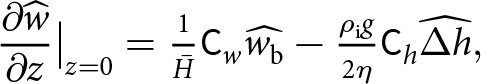


where we have defined the functions

(15)
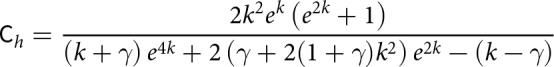

(16)
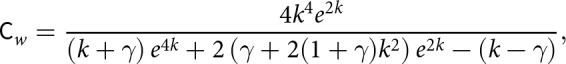

with 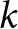 being the wavevector magnitude normalised by the background ice thickness 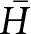. In Eqns. (15)-(16), we have introduced a nondimensional parameter that arises from the sliding law,
(17)
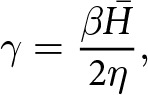


which relates all of the fundamental parameters that characterise the base state. The magnitudes of the functions 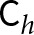 and 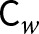 decay at larger values of the sliding parameter 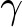, which leads to diminished viscous stresses at the base (Fig. 3a,b).

We combine Eqns. (13) and (14) to obtain a formula for the Fourier-transformed effective pressure,
(18)
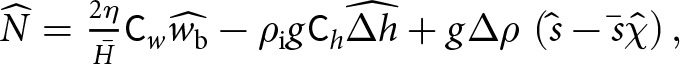


where we have assumed that the basal surface perturbation vanishes outside the lake boundary (
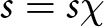
). The potential correlation between the effective pressure and the basal vertical velocity in (18) is analogous to the bending of an elastic beam (Evatt and Fowler, 2007). In particular, upward motion (
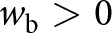
) can result in compression (
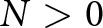
) at the base and downward motion can result in tension, although the precise level of correlation depends on the magnitude of the viscous term relative to the elevation-dependent terms. In Appendix C, we show that the opposite behaviour arises where the basal vertical velocity is proportional to the negative effective pressure in the limit of steady creep, which is consistent with commonly used creep closure laws for subglacial channels and other drainage elements (Nye, 1976; Hewitt, 2011). A simple, alternative interpretation relates the effective pressure to dynamic thickness changes, which are calculated implicitly with this method (Appendix B).

Following previous work, we obtain the basal vertical velocity 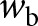 from elevation-change data 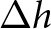 with an inverse method that follows the linearised approach taken herein (Stubblefield and others, 2023a). The ice-base elevation 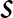 is determined up to an initial condition 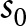 by
(19)
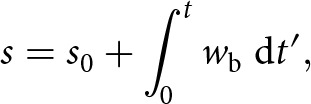


where we have neglected a small advective component from the background flow under the assumption that the ice–water interface motion is caused by the basal vertical velocity from water-volume changes (Stubblefield and others, 2023a). We can calculate the effective pressure from Eqn. (18) after specifying the initial conditions and auxiliary parameters (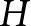, 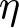, 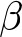, 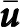) that the inverse method requires, where 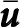 is the mean ice surface velocity in the base state (Stubblefield and others, 2023a). We provide a simplified analysis of Eqn. (18) in the following section for illustration, while a complete description of the inverse method is provided by Stubblefield and others (2023a).

## 3. Analysis

Before applying the estimation method to Antarctic subglacial lakes (Fig. 2), we first analyse the effective pressure 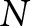 (Eqn. 18) in relation to the basal vertical velocity 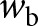. We then explore a synthetic example that falls within the parameter regime of the Antarctic subglacial lakes.

### 3.1. Scaling

First, we scale Eqn. (18) to facilitate the analysis below. For simplicity, we refrain from renaming the nondimensional variables. We let 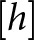 be the elevation anomaly scale (1 m) and 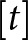 be the observational time scale (1 yr). We scale the map-plane coordinates 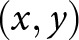 by 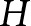, the effective pressure by 
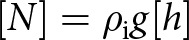
, and the vertical velocity by 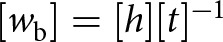. With this scaling, Eqn. (18) becomes
(20)
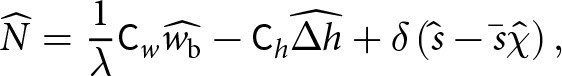


where we have defined the flotation factor 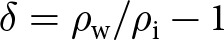. The parameter 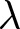 is defined by the ratio
(21)
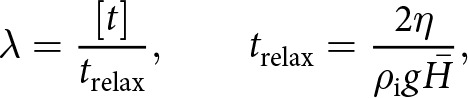


where 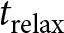 is the timescale for viscous relaxation (decay) of topography (Turcotte and Schubert, 2014; Stubblefield and others, 2021a; 2023a). The scaling in Eqn. (20) shows that the precise definition of the lake boundary is of lesser importance in determining the effective pressure since it only manifests in the smaller, hydrostatic term that is multiplied by the flotation factor. However, careful consideration of the lake boundary is important for determining where the underlying approximations are valid. Additionally, any suspended sediment in the water column could potentially increase the fluid density, making the hydrostatic term more important.

### 3.2. Closure relations

Next, we examine relations between the effective pressure and basal vertical velocity, which are related in subglacial hydrology models through creep closure laws (e.g., Nye, 1976; Evatt, 2015; Meyer and others, 2016). We outline how our formulation reduces to a similar form as the previously derived closure laws under certain simplifying assumptions in Appendix C. For simplicity, we assume here that there is no ice advection in the background state (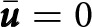) and no initial surface perturbations (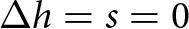 at 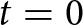). The general case has been previously covered (Stubblefield and others, 2023a). Under these assumptions, the elevation change is related to the basal vertical velocity by
(22)
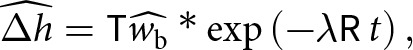


where 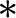 denotes convolution over time (Appendix A).

In Eqn. (22), 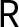 describes viscous decay of surface topography (Fig. 3c) while 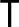 is a base-to-surface transfer function (Fig. 3d). These functions depend on the wavenumber 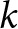 and nondimensional sliding parameter 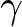 via

(23)
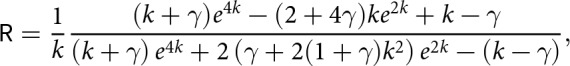

(24)
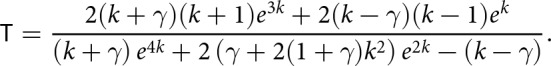

The functions (23)-(24) are rewritten slightly from previous work due to the scaling adopted herein (Stubblefield and others, 2023a). The elevation-change formula (22) provides the basis for the least-squares inverse method for obtaining the basal vertical velocity (Stubblefield and others, 2023a). Substituting the relations (19) and (22) into Eqn. (20), we obtain
(25)
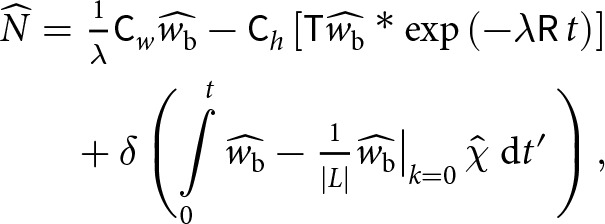


which provides a direct relation between the effective pressure and basal vertical velocity. In the limit of steady creep, Eqn. (25) reduces to a similar form relative to previous closure laws, where the closure rate is proportional to the negative effective pressure (Appendix C).

### 3.3. Sinusoidal oscillations

As a semi-analytical example, we assume that the basal vertical velocity oscillates in time according to
(26)
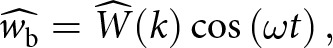


where 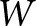 denotes the spatial pattern of the basal vertical velocity anomaly. We assume that the lake is radially symmetric with radius 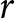 and surface area 
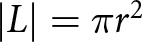
, which implies that the indicator function 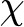 transforms to
(27)
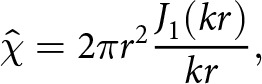


where 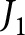 is the order-one Bessel function of the first kind.

We insert (26)-(27) into Eqn. (25), calculate the integrals and neglect an exponential decay term (proportional to 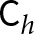) to obtain the long-time behaviour (
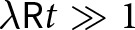
)

(28)
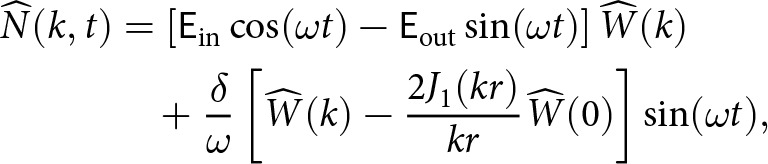

(29)
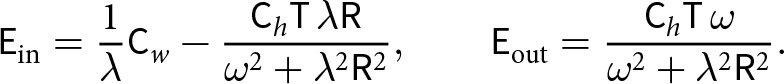

**Figure 4.**
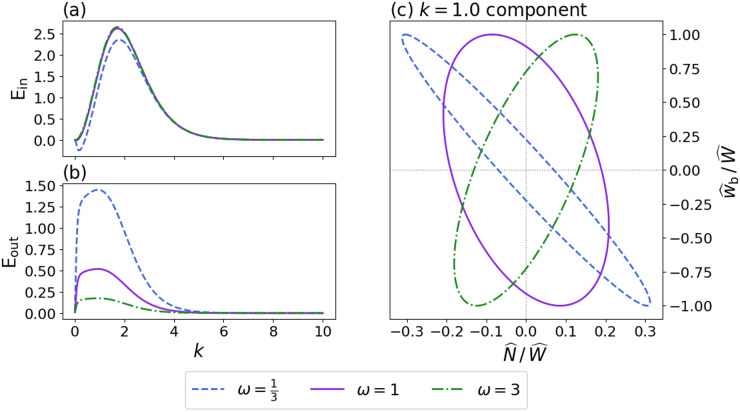
(a) In phase component 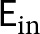 and (b) out-of-phase component 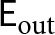 of the effective pressure spectrum (28) for different oscillation frequencies 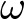. The nondimensional parameters are set to 
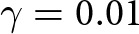
 and 
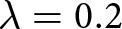
. (c) Effective pressure spectrum versus vertical velocity spectrum (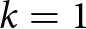 component), normalised by the spectral amplitude of the vertical velocity 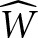. For this value of 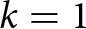, we set the long-wavelength term to 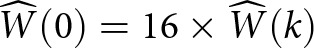 in Eqn. (28), which corresponds to the Gaussian-shaped anomaly in Figure 5.

We plot the in-phase component 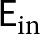 and out-of-phase component 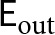 for various oscillation frequencies 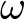 in Figure 4. The in-phase component 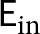 decreases as the oscillation frequency 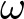 decreases. For all oscillation frequencies, the in-phase component decays to zero at short wavelengths and in the long-wavelength limit (Fig. 4a). The out-of-phase component 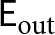 increases at slower oscillation frequencies (Fig. 4b). The spectra of the effective pressure and basal vertical velocity can be positively correlated, negatively correlated or uncorrelated, depending on the oscillation frequency and spatial wavenumber (Fig. 4c).

### 3.4. Synthetic example

**Figure 5.**
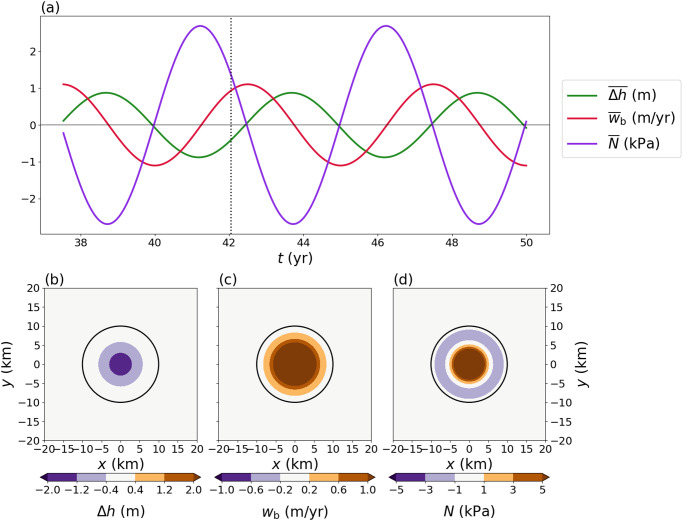
Synthetic example with nondimensional parameters 
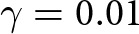
 and 
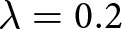
. (a) Time series of the mean elevation change, basal vertical velocity and effective pressure over the lake. (b)–(d) Map-plane plots of elevation change, basal vertical velocity and effective pressure at the time noted by the dashed vertical line in (a). The lake boundary is shown by the black circle.

To translate the preceding analysis to physical space, we consider a synthetic example with a Gaussian-shaped basal vertical velocity that exhibits sinusoidal oscillations in time (Fig. 5). In dimensional terms, the basal vertical velocity 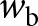 has a width of 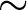
20 km, amplitude of 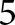 m yr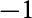 and oscillation period of 5 yr. While the relation between the effective pressure 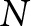 and basal vertical velocity 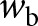 depends on the nondimensional parameters, we consider the values 
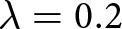
 and 
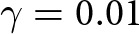
, which fall within the range of values for the Antarctic subglacial lakes discussed below (Table 1). The effective pressure is influenced by both the elevation-change anomaly (Fig. 5b) and the basal vertical velocity (Fig. 5c), but can be more strongly correlated with one over the other, depending on the parameter values. For these parameters, the mean effective pressure is negatively correlated with the elevation-change anomaly over time (Fig. 5a). Due to the combined influence of the basal velocity and elevation change, positive and negative values of the effective pressure can exist within the lake boundary simultaneously. For example, a ring-shaped region of negative effective pressure forms near the boundary of the lake at the start of the filling stages (Fig. 5d). Similar behaviour with the opposite sign can occur during the draining stages.

**Table 1.** Main parameters used in calculating the effective pressures of the Antarctic subglacial lakes (Figure 2). Data sources are provided in the ‘Data availability’ statement. The ‘Data’ section in the main text describes pre-processing of the elevation-change data and estimation of the ice-flow parameters (viscosity and basal drag). The off-lake pressure estimates 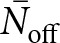 are defined in Eqn. (30). The nondimensional parameters are defined by 
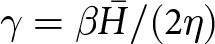
 (Eqn. 17) and 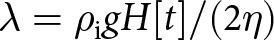 (Eqn. 21) where the observational timescale is 
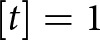
 yr. Parameter values are multiplied by the amount specified in the ‘units’ column.

Parameter	units	MSL	Mac1	David_s1_	Byrd_s10_
*Physical*					
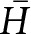 : ice thickness	m	1007	924	1991	2678
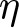 : ice viscosity	Pa s ( 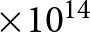 )	4.7	5.0	11.6	60.0
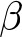 : basal drag coefficient	Pa s m 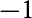 ( 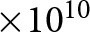 )	0.97	0.81	7.2	12.2
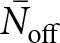 : off-lake pressure estimate	kPa	5.270	0.003	−0.937	0.124
*Nondimensional*					
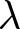 : 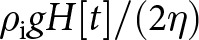	–	0.302	0.262	0.242	0.063
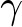 : 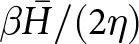	–	0.010	0.008	0.061	0.027

**Figure 6.**
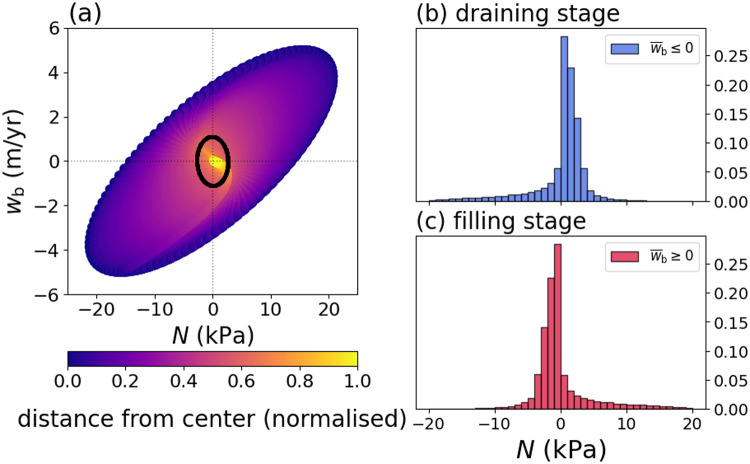
(a) Basal vertical velocity 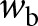 versus the effective pressure 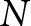 in the synthetic example (Figure 5) for different values of 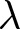. The nondimensional parameters are set to 
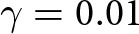
 and 
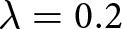
. The colours of the points show the distance from the centre of the lake normalised by the distance to the boundary. The black ellipse corresponds to the spatial mean over the lake at each point in time. (b) Histogram of the effective pressure during the draining stages (
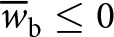
) normalised by the total number of spatiotemporal points. (c) Histogram of the effective pressure during the filling stages (
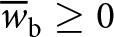
) normalised by the total number of spatiotemporal points.

The largest magnitudes of 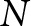 and 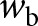 occur near the centre of the lake where the deformation is largest, while the mean behaviour over the lake has a smaller magnitude that more closely corresponds to the behaviour near the lake boundary (Figs. 5 and 6a). In particular, the mean values of 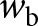 and 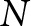 over the lake show a weaker correlation than the pointwise behaviour near the centre of the lake (Fig. 6a).

We compare histograms of the effective pressure for all spatiotemporal points during the draining stage (
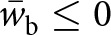
) and the filling stage (
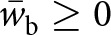
) to highlight the bulk behaviour of the system (Fig. 6b-c). The effective pressure distribution has a peak around 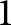 kPa during draining stages (
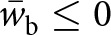
), accompanied by a long tail of negative values (Fig. 6b). During filling stages (
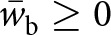
), the effective pressure distribution has a peak around 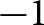 kPa that is accompanied by a long tail of positive values (Fig. 6c). For these parameters (
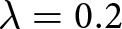
 and 
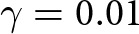
), the effective pressure falls within 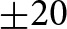 kPa, which is a relatively small deviation from flotation (
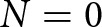
).

## 4. Antarctic examples

### 4.1. Data

We use Eqn. (18) to calculate the effective pressure 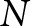 from the elevation-change anomalies 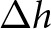 and the basal vertical velocity inversions 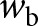 over several Antarctic subglacial lakes (Fig. 2). The preprocessing of the elevation-change data and the inversion for the basal vertical velocity are described in detail by Stubblefield and others (2023a). We use the ICESat-2 ATL15 L3B Gridded Antarctic and Arctic Land Ice Height Change (Version 4) data product (Smith and others, 2024) to obtain elevation-change anomalies over the lakes by removing any regional thinning or thickening trend (Stubblefield and others, 2023a). We linearly interpolate the ATL15 data onto the fine spatiotemporal grid of the inverse method, which could produce errors in the pressure estimates if lake activity occurs more rapidly than the temporal resolution of ICESat-2 (91-day repeat cycle).

Removing any regional thickness-change trend is necessary for the inverse method and coincides with the assumption of flotation (
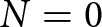
) in the base state. To provide an independent point of comparison for the results, we use the approximation (B.1) that relates effective pressure to dynamic thickness changes (Appendix B) to estimate an ambient effective pressure via
(30)
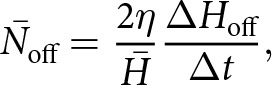


where 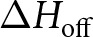 is the off-lake thickness change over the duration 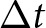 (5 years). We compute the off-lake thickness change by taking the spatial average of the ATL15 elevation-change product over all points that are at a distance greater than 80% from the centroid of the lake to the boundary of the computational domain (Stubblefield and others, 2021a). In the estimate (30), we have assumed a slip ratio of one and that all thickness changes arise from ice dynamics rather than snow accumulation (see Appendix B); removing any contributions from snow accumulation or assuming a smaller slip ratio would decrease the magnitude of the off-lake pressure estimates. We find that these estimates are at most only a few kilopascals in magnitude (Table 1), which is consistent with our results and underlying assumptions.

To invert the elevation-change data, we require the ice thickness 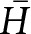, ice viscosity 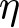, basal drag coefficient 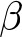 and horizontal ice velocity 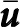 that are associated with the background ice-flow state over the lakes (Fig. 1). We obtain horizontal surface velocity from the MEaSUREs Phase-Based Antarctic Ice Velocity Map (Version 1) (Mouginot and others, 2019a; 2019b) and ice thickness from MEaSUREs BedMachine Antarctica (Version 3) (Morlighem and others, 2020; Morlighem, 2022). The basal drag and ice viscosity are estimated with the Ice-sheet and Sea-level System Model (shelfy-stream approximation) by inverting the surface-velocity data (Morlighem and others, 2010; Larour and others, 2012). The ice flow-law coefficient is estimated by providing the empirical relation from Cuffey and Paterson (2010) (with a flow-law exponent of 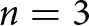) with an approximate depth-averaged temperature between the melting point and the surface temperature, which is obtained from the Modern-Era Retrospective Analysis for Research and Applications, version 2 (MERRA-2) climate reanalysis (Global Modeling and Assimilation Office (GMAO), 2015; Gelaro and others, 2017). All ‘background’ values are obtained by averaging the data 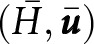 and modelled variables 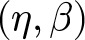 over a square region (60 km 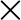 60 km) surrounding the lake (Stubblefield and others, 2023a). Sources for all datasets and the code repository are provided in the ‘Data availability’ statement. Parameter values for each lake are reported in Table 1.

The Antarctic subglacial lakes that we explore here have been the subject of many previous studies: Mercer Subglacial Lake beneath the confluence of the Whillans Ice Stream and Mercer Ice Stream (Fricker and others, 2007; Fricker and Scambos, 2009; Siegfried and others, 2016); Mac1 beneath MacAyeal Ice Stream (Fricker and others, 2010; Siegfried and Fricker, 2021); Byrd_s10_ beneath Byrd Glacier (Smith and others, 2009; Wright and others, 2014); and David_s1_ (sometimes referred to as D2) beneath David Glacier in East Antarctica (Smith and others, 2009; Lindzey and others, 2020; Malczyk and others, 2023). These lakes cover a wide range of physical parameters arising from different ice flow regimes across East Antarctica and West Antarctica (Fig. 2). In particular, the lakes display a variety of combinations of the nondimensional parameters 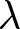 and 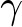 (Table 1). We first discuss the effective pressure estimates for each lake and then compare the behaviours between the lakes. Lake boundaries derived from elevation-change anomalies for these lakes are provided by Siegfried and Fricker (2018); we compare the altimetry-derived boundaries to the spatial extent of the effective pressure estimates below. As noted in the description around Eqn. (20), the particular choice of the lake boundary does not substantially influence the calculated effective pressure but is of primary importance in deciding where the calculation is valid.

### 4.2. Mercer Subglacial Lake

Mercer Subglacial Lake (MSL) exists beneath an ice thickness of 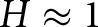 km at the confluence of the Whillans Ice Stream and Mercer Ice Stream along the Siple Coast of West Antarctica (Fig. 2). MSL has filled and drained repeatedly since the beginning of the ICESat era (Fricker and others, 2007; Fricker and Scambos, 2009; Siegfried and others, 2016; Siegfried and Fricker, 2018; 2021). The estimated ice viscosity (
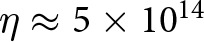
 Pa s) is an order of magnitude smaller than the East Antarctic lakes considered herein. The basal drag coefficient (
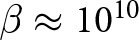
 Pa s m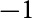) is slightly larger than Mac1, but the same order of magnitude or smaller than the East Antarctic lakes (Table 1).

**Figure 7.**
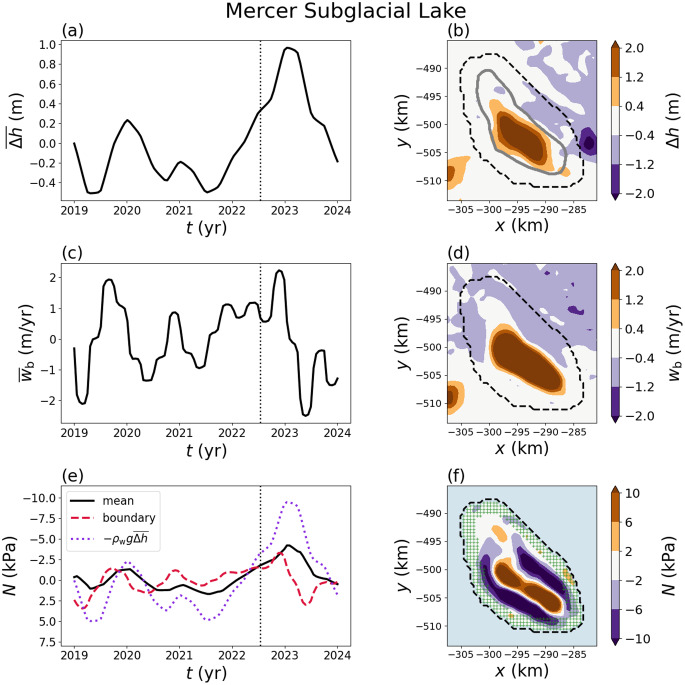
Elevation change (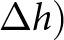, basal vertical velocity inversion (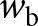) and effective pressure (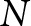) for Mercer Subglacial Lake. (a) Time series of the mean value of the elevation change over the lake. (b) Map–plane contour plot of the elevation change at the time shown by the vertical dashed line in (a). The dashed black line shows the boundary selected for calculating the effective pressure while the solid grey line shows the boundary from Siegfried and Fricker (2018). (c) Time series of the mean basal vertical velocity and (d) map–plane plot at the time shown by the vertical dashed line. (e) Time series of the mean effective pressure (solid), effective pressure within 2 km of the boundary (long dashed) and the reference pressure 
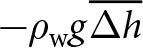
 (short dashed). (f) Map–plane plot of the effective pressure. The green hatched region corresponds to the values used to estimate the effective pressure near the lake boundary.

We estimate the effective pressure from the elevation-change anomaly and basal vertical velocity (Fig. 7). Since the beginning of the ICESat-2 period, the elevation-change data show that MSL completed a multi-peaked drainage event and has more recently begun to refill. The mean effective pressure in the lake, 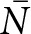, was small but positive (up to 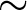
0.5 kPa) during the draining stage and has become negative during the filling stage as the ice column is lifted upwards (Fig. 7c). Maps of the elevation-change anomaly, basal vertical velocity and effective pressure during the filling stage highlight the spatial variability in the system (Fig. 7b,d,f). We find that the effective pressure has both regions of positive and negative effective pressure, which results from the combined influence of the basal vertical velocity and elevation change (Fig. 7f). In particular, the centre of the lake has a positive effective pressure that is surrounded by a ring of negative effective pressure that forms as the lake refills. The same type of ringed structure is found in the synthetic example (Fig. 5). We consider the possible consequences of this behaviour in the discussion. The altimetry-derived lake boundary from Siegfried and Fricker (2018) closely corresponds to the spatial extent of the effective pressure anomaly over MSL.

### 4.3. Mac1

**Figure 8.**
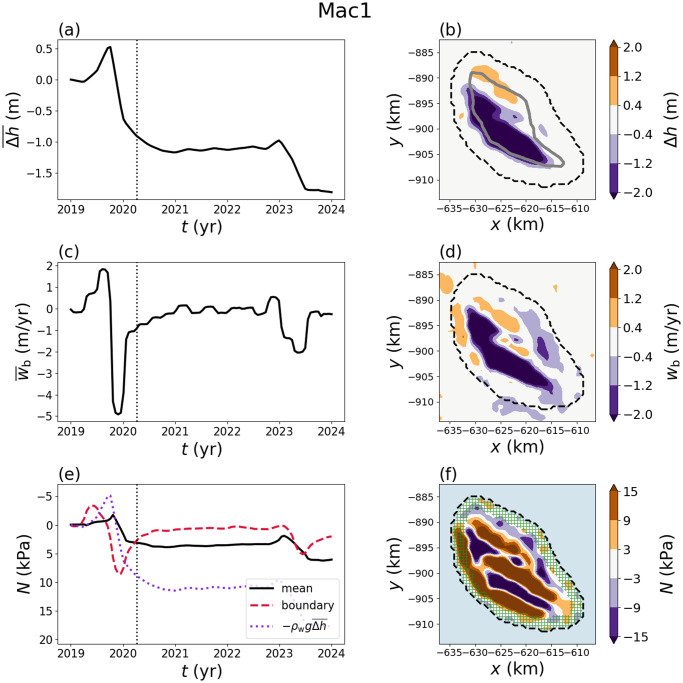
Elevation change (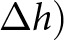, basal vertical velocity inversion (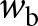) and effective pressure (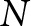) for Mac1. (a) Time series of the mean value of the elevation change over the lake. (b) Map–plane contour plot of the elevation change at the time shown by the vertical dashed line in (a). The dashed black line shows the boundary selected for calculating the effective pressure while the solid grey line shows the boundary from Siegfried and Fricker (2018). (c) Time series of the mean basal vertical velocity and (d) map–plane plot at the time shown by the vertical dashed line. (e) Time series of the mean effective pressure (solid), effective pressure within 2 km of the boundary (long dashed) and the reference pressure 
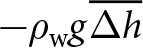
 (short dashed). (f) Map–plane plot of the effective pressure. The green hatched region corresponds to the values used to estimate the effective pressure near the lake boundary.

Subglacial lake Mac1 lies beneath the MacAyeal Ice Stream and has been observed to fill and drain repeatedly since the beginning of the ICESat era (Fricker and others, 2010; Siegfried and Fricker, 2021). The physical setting of Mac1 is similar to MSL with an ice thickness of 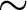
1 km and estimated viscosity on the order of 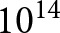 Pa s. However, the basal drag coefficient is estimated to be slightly smaller (Table 1). These physical parameters lead to smaller values of the nondimensional parameters 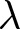 and 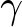 relative to MSL. The beginning of ICESat-2 observations coincided with a quiescent period followed by a draining event with a prolonged period of lowstand that lasted approximately two years (Fig. 8). The mean effective pressure was 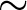
3 kPa in the lake after the draining event. More complex behaviour is seen in the spatial variability during the draining event where the effective pressure has positive and negative regions simultaneously (Fig. 8d). These complex patterns arise from thickness changes as the ice dynamically adjusts after the drainage event (Appendix B). The effective pressure showed a larger spatial extent than the altimetry-derived lake boundary during the draining event. Mac1 showed a second subsidence event with additional oscillations in the effective pressure during 2023–24.

### 4.4. David_s1_

**Figure 9.**
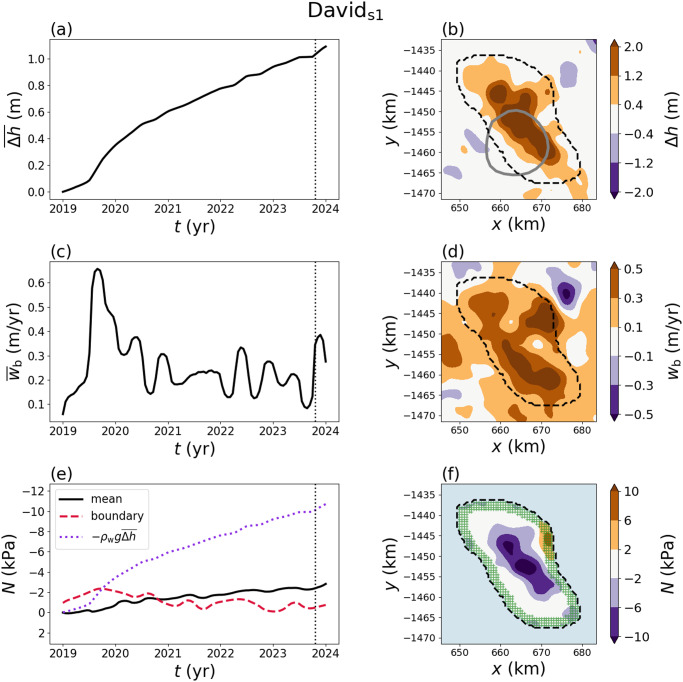
Elevation change (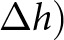, basal vertical velocity inversion (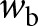) and effective pressure (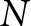) for David_s1_. (a) Time series of the mean value of the elevation change over the lake. (b) Map–plane contour plot of the elevation change at the time shown by the vertical dashed line in (a). The dashed black line shows the boundary selected for calculating the effective pressure while the solid grey line shows the boundary from Siegfried and Fricker (2018). (c) Time series of the mean basal vertical velocity and (d) map–plane plot at the time shown by the vertical dashed line. (e) Time series of the mean effective pressure (solid), effective pressure within 2 km of the boundary (long dashed) and the reference pressure 
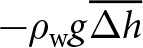
 (short dashed). (f) Map–plane plot of the effective pressure. The green hatched region corresponds to the values used to estimate the effective pressure near the lake boundary.

Subglacial lake David_s1_ lies beneath David Glacier, which feeds the Drygalski Ice Tongue in East Antarctica. David_s1_ showed activity during the ICESat era, along with several other lakes beneath David Glacier (Smith and others, 2009, referred to as lake D2 therein). Subsequent activity has been characterised by an overall upward trend in the elevation change over the lake (Lindzey and others, 2020; Malczyk and others, 2023). The physical setting in East Antarctica is characterised by a thicker ice cover of 
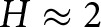
 km and higher viscosity (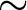
10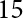 Pa s) relative to the examples from West Antarctica (MSL and Mac1). The basal drag coefficient is an order of magnitude larger than the examples from West Antarctica, corresponding to slow ice flow speeds (Table 1). David_s1_ has continued to fill during the ICESat-2 period, causing an increasingly negative effective pressure within the lake (Fig. 9). The effective pressure and the basal vertical velocity show correlated oscillations over time at this higher viscosity value (Fig. 9c,e). These oscillations correspond to fluctuations in the *rate* of elevation change that manifest as slope changes in the elevation-change timeseries (Fig. 9a). The effective pressure shows a similar spatial extent to the elevation-change anomaly, although the spatial footprint appears to have changed significantly since the ICESat-era drainage event (Smith and others, 2009; Siegfried and Fricker, 2018; Lindzey and others, 2020). We revisit this discrepancy between the ICESat-era outline and the current boundary in the discussion.

### 4.5. Byrd_s10_

**Figure 10.**
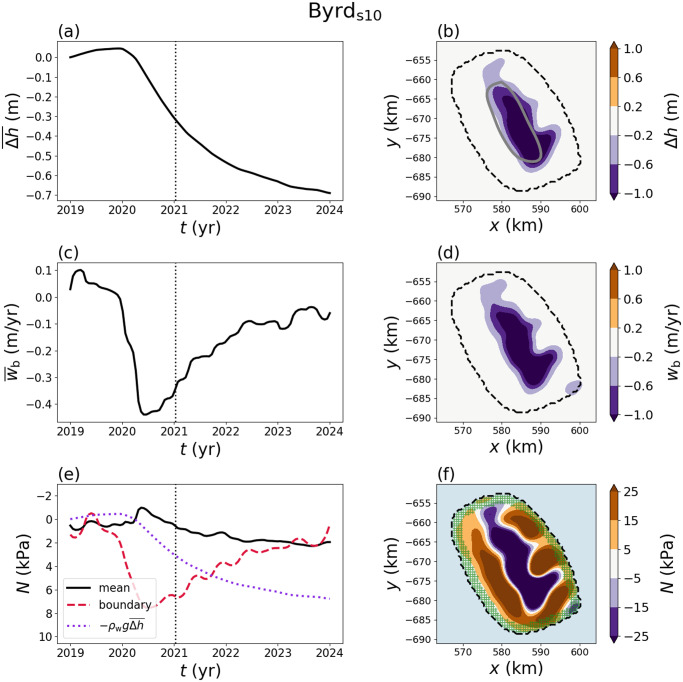
Elevation change (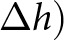, basal vertical velocity inversion (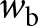) and effective pressure (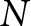) for Byrd_s10_. (a) Time series of the mean value of the elevation change over the lake. (b) Map–plane contour plot of the elevation change at the time shown by the vertical dashed line in (a). The dashed black line shows the boundary selected for calculating the effective pressure while the solid grey line shows the boundary from Siegfried and Fricker (2018). (c) Time series of the mean basal vertical velocity and (d) map–plane plot at the time shown by the vertical dashed line. (e) Time series of the mean effective pressure (solid), effective pressure within 2 km of the boundary (long dashed) and the reference pressure 
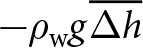
 (short dashed). (f) Map–plane plot of the effective pressure. The green hatched region corresponds to the values used to estimate the effective pressure near the lake boundary.

Subglacial lake Byrd_s10_ beneath Byrd Glacier in East Antarctica showed activity during the ICESat period (Smith and others, 2009). The physical setting in East Antarctica is characterised by a thicker ice cover of 
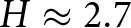
 km and higher viscosity (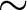
10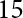 Pa s) relative to the examples from West Antarctica (MSL and Mac1). However, the estimated basal drag coefficient is an order of magnitude smaller than for David_s1_ (Table 1). Byrd_s10_ was quiescent at the beginning of the ICESat-2 period and has drained over the course of several years (Fig. 10a). The draining is associated with an elevated mean effective pressure within the lake. However, there is a large region of negative effective pressure that forms near the centre of the lake (Fig. 10f). Negative effective pressures arise when tensile viscous stresses dominate Eqn. (18) during draining events (
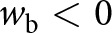
) with higher ice viscosity. The spatial extent of the effective pressure is much larger than the elevation-change anomaly due to these high tensile viscous stresses, which shows that subglacial pressure perturbations can extend beyond altimetry-derived lake outlines (Fig. 10d). In the discussion, we describe the consequences of a larger effective pressure footprint and how this phenomenon can be illustrated analytically via a Green’s function analysis (Appendix C).

### 4.6. Effective pressure distributions

**Figure 11.**
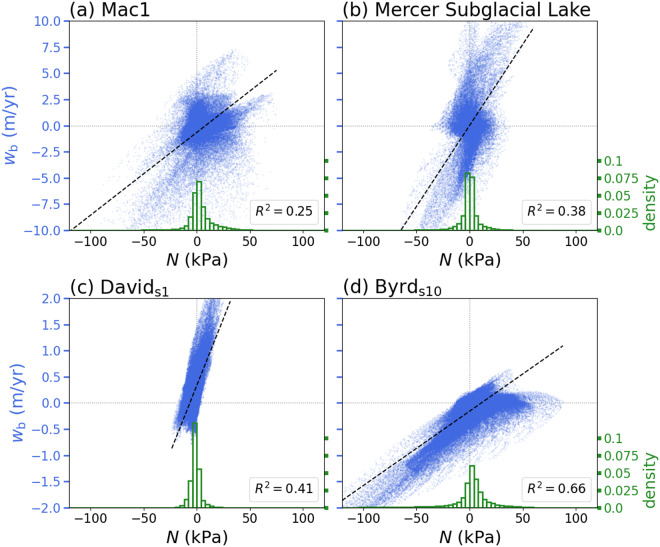
Basal vertical velocity 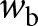 versus the effective pressure 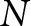 for the Antarctic subglacial lakes shown in Figures 7-10. Each point within the lake boundary is plotted for each point in time (blue points). Linear regressions are shown by the dashed black lines. Green histograms show effective pressure distributions normalised by the total number of points and the bin width.

The preceding examples show that the effective pressure can be more strongly correlated with the basal vertical velocity or the elevation-change anomaly, depending on the physical properties of the ice and bed surrounding the subglacial lake (Figs. 7-10). We examine the relations between 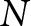 and 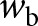 for each lake by plotting the values for all spatiotemporal points within the lake boundaries (Fig. 11). The West Antarctic lakes (MSL and Mac1) and David_s1_ show only weak correlations between the effective pressure and the basal vertical velocity while Byrd_s10_ shows a stronger correlation (Fig. 11). The stronger correlation is due to the higher estimated ice viscosity at Byrd_s10_, which also results in effective pressures that reach larger magnitudes. Histograms of the effective pressure for all spatiotemporal points within the lake boundaries show that the West Antarctic lakes and David_s1_ are more closely clustered around flotation (
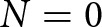
) while Byrd_s10_ has a wider and shorter distribution (Fig. 11). The effective pressures are generally on the order of 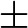
10 kPa, although they can locally reach values of 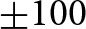 kPa.

## 5. Discussion

Here, we have developed a method for estimating effective pressures in subglacial lakes from altimetry data and applied the method to a collection of active subglacial lakes in Antarctica. We have shown that the effective pressure of a subglacial lake can be correlated with either the basal vertical velocity or the elevation-change anomaly. In particular, at higher values of the ice viscosity, we find that the effective pressure is more strongly correlated with the basal vertical velocity. While the subglacial lakes considered herein tend to have effective pressures within 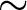
10 kPa of flotation, locally elevated effective pressures can arise when the viscosity is large (e.g., Fig. 11). These results highlight the importance of accounting for viscous stresses when estimating the effective pressure and the potential difficulty of interpreting the basal stress state from elevation-change anomalies alone. Moreover, the computational efficiency of the method could facilitate quantifying uncertainties arising from the ice viscosity, which varies over orders of magnitude, as well as incorporating other constraints, like lake depth, in a statistical framework.

**Figure B1.**
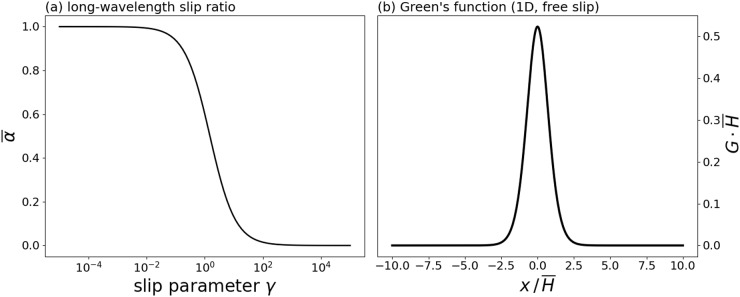
(a) Linearised long-wavelength slip ratio 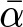 (Eqn. B.4) as a function of the slip parameter 
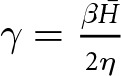
. The limit 
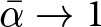
 as 
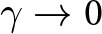
 corresponds to free slip while 
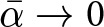
 as 
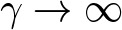
 corresponds to no slip. (b) One-dimensional Green’s function 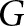 (Eqn. C.2), normalised by 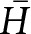, in the limit of vanishing basal drag (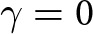).

While we focused on how viscosity influences effective pressure estimates, the basal drag coefficient can also influence the results to a lesser degree. We showed that the effective pressure can be approximated by the logarithmic strain rate of the ice column multiplied by the ice viscosity and a slip ratio (Appendix B). In slow-flowing regions, the slip ratio could be small enough to drive the effective pressure towards flotation, but this may be counteracted by higher ice viscosity. The results herein are not strongly influenced by basal drag coefficient variability because all examples had a nondimensional sliding parameter of the same magnitude, resulting in approximately equal slip ratios (Table 1; Fig. B1a).

The subglacial lake effective pressures found here are small compared to other drainage elements such as channels and cavities where the effective pressure can reach megapascals in magnitude, which could have important consequences for the drainage system (e.g., Dow and others, 2022; Sommers and others, 2023). For example, negligible effective pressure near the boundary of a subglacial lake could inhibit closure of adjacent drainage elements, which in turn might facilitate shoreline migration via melting or sediment erosion (Evatt and others, 2006). The smallness of the subglacial lake effective pressure relative to neighbouring drainage elements could also generate large gradients in the hydraulic potential that influence the magnitude of water flow. Conversely, elevated effective pressures due to higher viscous stresses may result in a diminished hydraulic gradient, which could hinder water capture, depending on the pressure in the surrounding drainage system. The effective pressure of a subglacial lake being near flotation could therefore be important for influencing flooding events and water capture, although more comprehensive hydrology modelling would be needed to further constrain these dynamics.

The behaviour near the lake boundary is important in relation to potential interactions with the surrounding drainage system. We found a consistent spatial pattern where the effective pressure changes sign near the lake boundary, which arises from the combined influence of the elevation-change anomaly and basal velocity on dynamic thickness changes (Appendix B). For example, the effective pressure became negative near the boundary of Mercer Subglacial Lake when it began to refill, while positive effective pressures existed near the centre of the lake from the lowstand (Fig. 7). Negative effective pressures near the boundary of a lake could cause detachment of ice from the base and shoreline migration, or inhibit creep closure of a subglacial channel that drains the lake, setting the stage for future flooding events (Evatt and others, 2006; Fowler, 2009; Stubblefield and others, 2019, 2021b). On the other hand, we found positive effective pressures developing during draining events near the boundaries of Mac1 and Byrd_s10_, which could be associated with channel closure and the eventual cessation of flooding.

We also found that the areal extent of the effective pressure can extend beyond the spatial extent of the elevation-change anomaly due to viscous stresses within the ice (Fig. 10). To further analyse this behaviour, we derived a Green’s function for the simplified problem of steady creep and found that the effective pressure depends on the basal vertical velocity over zone that is roughly five ice thicknesses wide (Appendix C, Fig. B1). The presence of pressure perturbations that extend beyond the altimetry-based lake outline raises the issue of how subglacial lake boundaries should be identified. For example, it is difficult to determine if water that exists outside of the altimetry-based outline should be considered part of the drainage system or part of the lake without further modelling to assess the hydraulic state.

In a similar vein, the results show that the current lake boundaries do not necessarily coincide with the previous ICESat-era elevation changes, suggesting that subglacial shorelines have potentially migrated relative to previous positions (Siegfried and Fricker, 2021). David_s1_ provides an example of this behaviour where water is ponding in a different area relative to the ICESat-era observations (Fig. 9). A detailed aerogeophysical survey over the David Glacier lake system showed that a minimum in the hydraulic potential existed north of the ICESat-era outline (Lindzey and others, 2020), which is consistent with the recent ICESat-2 elevation changes (Fig. 9). While theory and observations suggest that shorelines can migrate during volume-change events, incorporating these physics into subglacial hydrology models remains an open challenge (Siegfried and Fricker, 2021; Stubblefield and others, 2021a, 2021b).

The effective pressure estimation method developed herein only applies to subglacial lakes, which we have defined as drainage elements that have a minimal hydraulic potential and small slope at the ice base. Other drainage elements like subglacial channels, cavities or sheets do not generally satisfy these conditions (Flowers, 2015). While these other drainage elements do not produce localised elevation-change anomalies, which cause variations in basal drag through sliding relations that indirectly influence elevation-change patterns by modulating ice flow speeds. Connecting elevation changes to subglacial water flow through a broader array of drainage elements is an open challenge that can be addressed in future work with more comprehensive hydrology models (Werder and others, 2013; Sommers and others, 2018).

As the dynamics of subglacial lakes are closely coupled with the surrounding drainage system, the effective pressure estimates derived herein can be used to further constrain or validate subglacial hydrology models in drainage basins hosting subglacial lakes. Likewise, testing subglacial hydrology models against these estimates would help to further constrain transient changes in subglacial sliding. Future work should therefore focus on constraining hydrology models and ice-flow models with these effective pressure estimates.

## 6. Conclusions

Here, we have developed a method for estimating the subglacial effective pressure from elevation-change data above active subglacial lakes. We applied the method to a collection of Antarctic subglacial lakes that have shown activity during the ICESat-2 era and found that the effective pressure in these lakes tended to remain within a few tens of kilopascals of flotation. Our analysis suggests that higher viscous stresses are associated with greater deviations from flotation. Similarly, we found that the effective pressure can be more strongly correlated with either the elevation-change anomaly or the basal vertical velocity over time, depending on the magnitude of the viscous stresses at the base. The effective pressure estimates developed herein can be used to help validate subglacial hydrology models with altimetry data and further constrain the dynamics of the glacier bed.

## Data Availability

All data used in this study are openly available: ICESat-2 ATL15, Version 4 (https://doi.org/10.5067/ATLAS/ATL15.004), MEaSUREs Phase-Based Antarctica Ice Velocity Map, Version 1 (https://doi.org/10.5067/PZ3NJ5RXRH10), MEaSUREs BedMachine Antarctica, Version 3 (https://doi.org/10.5067/FPSU0V1MWUB6), MERRA-2 monthly mean surface temperature (https://doi.org/10.5067/AP1B0BA5PD2K), Subglacial lake inventory from Siegfried and Fricker (2018) (https://doi.org/10.5281/zenodo.4914107). The code used to produce the results is available as an archived repository (https://doi.org/10.5281/zenodo.17859686).

## Appendix A. Linear model derivation

A general formula for the vertical velocity that satisfies the linearised Stokes equations is

(A.1)

where  denotes the wavevector magnitude scaled by the background ice thickness  and the coefficients  are determined by the boundary conditions (e.g., Gudmundsson, 2003; Stubblefield and others, 2023b). We take the derivative of (A.1) and set  to obtain

(A.2)

The linear system for the coefficients  is

(A.3)

where  (Stubblefield and others, 2021a; Stubblefield, 2022). The first row in the system (A.3) represents the perturbed normal stress at the ice surface (), the second row represents the vanishing shear-stress at the ice surface, the third row represents a sliding law at the base () and the fourth row prescribes the basal vertical velocity  (cf. Stubblefield and others, 2023b). We obtain Eqn. (14) by solving the system (A.3) for the coefficients  and substituting them into Eqn. (A.2). To obtain the expression for the elevation-change anomaly (22), we substitute the coefficients  into the identity

(A.4)

and use the kinematic condition , which holds in the limit of no background advection (). In this way, we obtain , which is an ordinary differential equation for  with respect to time that is solved (after scaling) by Eqn. (22) (Stubblefield and others, 2023a).

## Appendix B. Relation to dynamic thickness changes

While the main text considers the effective pressure as a function of the elevation change and basal vertical velocity independently, there is a simple approximation in terms of dynamic thickness changes. For simplicity, we assume a constant slip ratio  defined by the approximation , where  denotes the depth-averaged horizontal ice velocity. In the absence of surface accumulation and basal melting, ice thickness evolves according to  with  denoting the material derivative (Greve and Blatter, 2009). We use the effective pressure Eqn. (10) and the slip ratio approximation to obtain

(B.1)

which relates the effective pressure to the logarithmic strain rate of the ice column. The approximation (B.1) could be applied to thickness-change data from autonomous phase-sensitive radio-echo sounding (ApRES) to provide an alternative, independent estimate of the effective pressure (e.g., Siegfried and others, 2023).

We confirm that the linearised formulation is consistent with the approximation (B.1) in the long-wavelength limit. In the linearised model, the full thickness, including the perturbations, is given by  so that thickness evolves according to . We use the identity for the elevation change from Appendix A () in Eqn. (18) and rearrange to obtain

(B.2)

In Eqn. (B.2), we have introduced the function

(B.3)

which is a smooth, decaying function of the wavevector magnitude . The function  plays the role of the slip-ratio in the long-wavelength limit and is also involved in steady-state creep closure (Appendix C). We define a linearised long-wavelength slip ratio  via

(B.4)

The linearised slip ratio  depends on the slip parameter  (Fig. B1a). Upon taking the long-wavelength limit of Eqn. (B.2), the last two terms vanish and we obtain

(B.5)

which relates the effective pressure to the engineering strain rate (linearised logarithmic strain rate) in agreement with the approximation (B.1).

## Appendix C. Closure laws during steady creep

Here, we outline how the effective pressure and basal vertical velocity are negatively correlated in the limit of steady creep. Closure laws for subglacial channels and other drainage elements assume a similar negative relationship (Hewitt, 2011; Evatt, 2015). Assuming steady state (i.e., ) and neglecting the small hydrostatic term, Eqn. (B.2) reduces to

(C.1)

where  is defined in (B.3). Taking the inverse Fourier transform of (C.1) yields a spatial convolution. During steady creep, the effective pressure can therefore be obtained from the spatial convolution of the negative closure rate () with a smooth filter. Deconvolving this relation to obtain a closure law of the form  would require some form of regularisation.

In the limits of vanishing basal drag () and one spatial dimension (i.e., the  direction), we obtain

(C.2)

The Green’s function  shows that the closure rate influences the effective pressure in a zone that is nearly five ice thicknesses wide (Fig. B1b). Therefore, the areal extent of the effective pressure can extend beyond the areal extent of the basal vertical velocity or the altimetry-derived lake outline. The correspondence with creep closure laws can be found by seeing that the long-wavelength limit (B.5) in steady state implies  which is a negative relation similar to closure laws for subglacial channels and other drainage elements.
